# Bioinspired Thermal Conductive Cellulose Nanofibers/Boron Nitride Coating Enabled by Co-Exfoliation and Interfacial Engineering

**DOI:** 10.3390/polym16060805

**Published:** 2024-03-14

**Authors:** Xinyuan Wan, Xiaojian Xia, Yunxiang Chen, Deyuan Lin, Yi Zhou, Rui Xiong

**Affiliations:** 1 State Grid Fujian Electric Research Institute, Fuzhou 350007, China; wanxinyuan89103@163.com (X.W.); xia.xiaojian@gmail.com (X.X.); rogerchen614@163.com (Y.C.); lindeyuan_fj@126.com (D.L.); 2 State Key Laboratory of Polymer Materials Engineering, Polymer Research Institute of Sichuan University, Chengdu 610065, China; yizhou@stu.scu.edu.cn

**Keywords:** cellulose nanofibers, boron nitride, layered structure, thermal conductive coating, mechanical properties

## Abstract

Thermal conductive coating materials with combination of mechanical robustness, good adhesion and electrical insulation are in high demand in the electronics industry. However, very few progresses have been achieved in constructing a highly thermal conductive composites coating that can conformably coat on desired subjects for efficient thermal dissipation, due to their lack of materials design and structure control. Herein, we report a bioinspired thermal conductive coating material from cellulose nanofibers (CNFs), boron nitride (BN), and polydopamine (PDA) by mimicking the layered structure of nacre. Owing to the strong interfacial strength, mechanical robustness, and high thermal conductivity of CNFs, they do not only enhance the exfoliation and dispersion of BN nanoplates, but also bridge BN nanoplates to achieve superior thermal and mechanical performance. The resulting composites coating exhibits a high thermal conductivity of 13.8 W/(m·K) that surpasses most of the reported thermal conductive composites coating owing to the formation of an efficient thermal conductive pathway in the layered structure. Additionally, the coating material has good interface adhesion to conformably wrap around various substrates by scalable spray coating, combined with good mechanical robustness, sustainability, electrical insulation, low-cost, and easy processability, which makes our materials attractive for electronic packaging applications.

## 1. Introduction

The urgent requirement of miniaturized, densified, and multi-functional electronics significantly increases the power density of electronics, leading to fast heat accumulation in a limited or confined space [1,2]. This rapidly increased temperature inevitably affects the service life, safety, reliability, and speed of the electronics, even causing severe equipment damage and major fires. To address this issue, progress has been made to develop thermally conductive materials that can effectively dissipate the accumulated heat. Metallic and nanocarbon materials, including aluminum, MXene, graphene, and carbon nanotube have been intensively incorporated into polymeric matrix for constructing flexible, light weight composites with high-performance thermal management capabilities [3,4,5]. Although these resulting composites exhibit outstanding thermal conductivity, they usually also possess high electrical conductivity [6,7,8], which easily cause undesired short-circuit problem in the application of electronics. On the other hand, to achieve efficient thermal management, a seamless interface between electronics and thermal conductive composites is strictly needed. However, the conformal integration of these pre-formed thermal conductive composites onto the electronics is difficult due to the poor interface adhesion, especially for some irregular surfaces. The addition of glue could solve the adhesion problem but introduces additional thermal resistance and cost. Therefore, it is desirable to develop a thermal conductive but electrical insulating composites coating that can be seamlessly assembled on various irregular objects.

Boron nitride (BN) not only has excellent thermal conductivity and electrical insulation property, but also has good chemical stability and oxidation resistance, making it a promising candidate as a nanofiller for constructing thermal conductive composite coating [9,10]. However, most BN-based composites coating still face the issues of low thermal conductivity and weak mechanical properties, due to the poor dispersion of conductive filler and lack of structure control, largely limiting their practical applications [11,12]. To realize high thermal conductivity and mechanical robustness, bioinspired structural hierarchy design is one of the most promising approaches to engineer the composites coating [13]. The most spectacular examples are nacre-like composites, using aligned micro/nanoplates in polymer matrix [14,15]. For instance, Han et al. report a strong nacre-mimetic BN/epoxy composite by using a bidirectional freezing technique, which can realize high thermal conductivity of 6.07 Wm^−1^K^−1^ combined with good electrical insulation performance [15]. Pan et al., took advantage of hot-pressing technique to construct brick and mortar-structured Ag-Al_2_O_3_ platelets/epoxy composites with a thermal conductivity of 6.71 Wm^−1^K^−1^ [16]. However, these techniques usually need complex processes, which are difficult to be apply in composite coating and constructing nanostructured composites coating with a combination of high thermal conductivity, mechanical robustness, and good adhesion is still a big challenge.

In nature, nacres utilize chitin nanofibers which are wrapped by proteins to glue CaCO_3_ microplates to form hierarchical layered structures [17]. The highly ordered architecture and favorable interfacial strength enable nacre to have an amazing combination of mechanical strength and toughness, with added brilliant iridescence [18]. In this study, we report a bioinspired high-performance thermal conductive coating by exfoliation/dispersion of BN nanoplate and cellulose nanofibers (CNFs) and multiple interfacial interaction engineering. We take advantage of sustainable 1D CNF and dopamine as the building blocks to mimic the combination of chitin and proteins. CNFs have a similar structure to chitin, but it is more widely available and stronger [19,20,21], while polydopamine (PDA) is a well-known mussel-inspired adhesive that mimics the adhesive properties of mussels, which can adhere to a variety of surfaces under wet and dry conditions [22]. CNFs and BN nanoplates are integrated together to form viscose ink through the simultaneous exfoliation/dispersion induced by the strong shearing force of pan mill. The subsequent incorporation of dopamine not only significantly enhanced the interfacial strength of the composites, but also enabled strong adhesion to various substrates as conformal coating. The resulting coating demonstrates thermal conductivity of 13.8 W/(m·K) combined with good mechanical properties because the uniform layered structure provides prolonged phonon pathways. Thus, such an outstanding combination of thermal conductivity, mechanical properties, electrical insulation, adhesion, sustainability, and scalable process make our composites promising candidates for advanced electronic packaging technology applications.

## 2. Materials and Methods

### 2.1. Materials

Bleached wood pulp was purchased from Dalian Yangrun Trading Co., Ltd., Dalian, China. 2,2,6,6-Tetramethylpiperidine-1-oxyl (TEMPO), sodium hydroxide (NaOH), and dopamine hydrochloride were purchased from Aladdin Industrial Co., Shanghai, China. Sulfuric acid, sodium bromide (NaBr), sodium hypochlorite (NaClO), and hydrochloric acid were purchased from Xilong Scientific Co., Ltd., Guangdong, China. BN powder was purchased from Zhengzhou Jiajie Chemical Products Co., Ltd., Zhengzhou, China. Tris-HCl buffer was purchased from Fuzhou Feijin Biotechnology Co., Ltd., Fuzhou, China.

### 2.2. TEMPO Oxidation of Cellulose Fibers

Briefly, cellulose bleached wood pulp (50 g) was added to distilled water (5000 mL) to obtain homogenous dispersion with vigorous stirring. Then, a pH meter was placed in the suspension to monitor its pH value. TEMPO (0.78 g), NaBr (5.113 g), and 11 wt % NaClO (322.7 g) were separately added in the above suspension. Under constant stirring, the 0.1 M HCl was used to adjust the pH of the system to maintain it at around 10. As the reaction proceeds, the pH of the solution decreases. To maintain the pH of the system, 0.1 M NaOH solution was added slowly. The reaction was terminated when there is no change of the pH in the system for at least 30 min. The as-prepared TEMPO oxidized cellulose slurry was thoroughly washed until neutral and isolated by sieve. After measuring the mass of fibers, the cellulose slurry was diluted to 2 wt % cellulose fiber suspension.

### 2.3. Co-Exfoliation/Dispersion of BN Nanoplate and Cellulose Nanofibers (CNFs)

Co-exfoliation/dispersion of BN nanoplate and cellulose nanofibers (CNFs) were carried out according to the previous study [23]. Under continuous stirring, different amounts of BN powder were suspended in 2000 mL cellulose fiber suspension. Then, the suspensions were milled by an ultra-fine friction grinder Supermass colloider (MKCA6-2, Masuko Sangyo Co., Ltd., Kawaguchi, Japan) at 1500 rpm to obtain homogeneous CNFs/BN nanoplates mixture. The initial pan gap distance was set as 0 μm which was the point of slight contact between the two pans to avoid blocking. The pan gap distance was set from large to small. The distance between the two pans was reduced from 0 μm to −10 μm, −50 μm, −100 μm, and −150 μm, respectively. The suspensions were treated 10 times at every position to obtain 5 wt % homogeneous CNFs/BN nanoplates mixture (BCNF). The mixture with different BN contents related to the total mass of the suspension were coded to be BCNF10, BCNF30, BCNF50, and BCNF70 for 10 wt %, 30 wt %, 50 wt %, and 70 wt %, respectively. For a better comparison, the solid content of all BCNF samples (10–70 wt %) are the same 5 wt %.

### 2.4. Self-Polymerisation of Dopamine

The self-polymerization of dopamine was carried out according to the reported works [24,25,26]. Typically, dopamine hydrochloride (0.5 g) was added to 100 mL Tris-HCl buffer (10 mM, pH 8.5) under vigorous stirring for 24 h at room temperature. Then, the mixtures were centrifuged (10,000 rpm, 10 min) and washed with distilled water 3 times. After drying under vacuum, the PDA solution was obtained through re-dispersion in water at the concentration of 5 mg/mL.

### 2.5. Preparation of Thermal Conductive Film and Coating

In a typical process, 10 mL PDA solution (5 mg/mL) was added to 19 g BCNF (5 wt %) under stirring to prepare thermal conductive composite slurries (CNFs/BN/PDA). The composites films were fabricated by vacuum filtration of the slurries on a cellulose filter membrane with a 0.45 μm pore size. The as-prepared films were dried at room temperature and peeled off from the filter membrane. The thickness of membrane could be controlled by adjusting the volume of CNFs/BN/PDA slurries. Here, the thickness was adjusted to 200 ± 20 μm. The thermal conductive coating was fabricated by spraying CNFs/BN/PDA slurries on different substrates. In the typical spraying process, the substrates were fixed on fixtures and sprayed with different substrates using a spray gun fitted with CNFs/BN/PDA slurries. Then, they were air dried at room temperature.

### 2.6. Characterization

The Micro/nano structures of BN powder, cellulose bleached wood pulp, CNFs/BN/PDA slurries, and films were characterized by field emission scanning electron microscope (SEM, Thermo Fisher Scientific, Shanghai, China (FEI, Apreo S HiVoc)). The morphologies of BCNF were characterized using a high-resolution transmission electron microscopy (TEM, Tecnai G2 F20 S-TWIN, Hillsboro, OR, USA) at 200 Kv. To study the rheological behavior of BCNF with different contents of BN, the rheological tests were performed by an advanced rotational rheometer (MCR302, Anton Paar, Graz, Austria). The stress–strain curves of the CNFs/BN/PDA films were obtained on the Instron 5966 universal testing machine (Instron, Boston, MA, USA) with a 1000 N load cell. The stress–strain curves of three identical spline patterns were repeated for samples with different BCNF ratios, and the error bars were obtained from the three sets of data. The thermal conductivity values (λ, W/m·K) of CNFs/BN/PDA films were characterized by a thermal constant analyzer (Hot Disk, 2500S, Goteborg, Switzerland). A pull-off method adhesion tester (BJZJ-M, Zhongjiaojianyi Testing Equipment Co., Beijing, China) was used to test the adhesion strength of CNFs/BN/PDA coatings on different substrates. The water contact angle of the coatings was characterized by an optical contact angle measuring instrument (KRUSS, DSA30, Hamburg, Germany).

## 3. Results and Discussion

**Figure 1:**
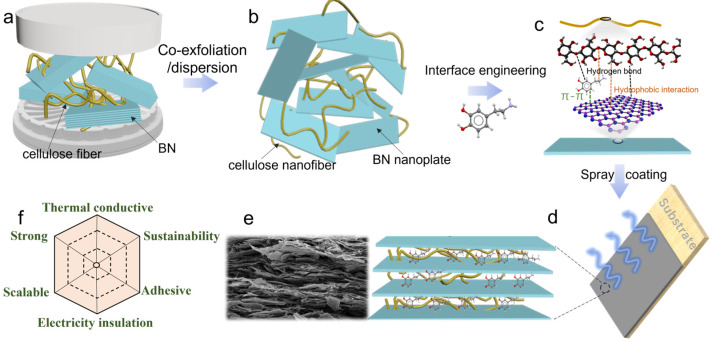
(**a**–**d**) The schematic illustration of the preparation of bioinspired thermal conductive composite slurries from BN, CNFs, and dopamine. (**e**) SEM image of cross section of composites coating. (**f**) The properties of the composites coating.

The schematic representation shown in Figure 1a–d illustrates the fabrication of the CNFs/BN/PDA thermal conductive composites ink. To facilitate the exfoliation and dispersion of CNFs, soft wood pulp has been pretreated using TEMPO oxidation, which selectively oxidizes the primary hydroxyl groups (C6) on the surface of cellulose fibers to negatively charged carboxyl groups [27]. The pretreated cellulose fibers were mixed with BN and subjected to pan mill to co- exfoliate and disperse CNFs/BN nanoplates mixture. During the process of co-exfoliation and dispersion, stacked large-size BN particles are peeled off into nanosheets, using the strong shear force of two pans. Meanwhile, the pretreated cellulose fibers also become nano fibers (CNFs). CNFs could inhibit the re-stacking and agglomeration of BN nanosheets through hydrogen bonding, hydrophobic interactions, and spatial site resistance, resulting in stable CNFs/BN slurries (Figure S1) [23]. However, the resulting slurries are difficult to apply onto various substrates due to poor adhesion. To achieve a wide range of adaptability to different substrates, 5 wt % PDA were added into the above slurries to regulate the interactions. The strong multiple interactions, including hydrogen bonding, hydrophobic interaction, and π-π stacking interactions, would greatly enhance both adhesion with substrates and internal interactions [28]. As shown in Figure S2, the Fourier Transform Infrared Spectrometer (FTIR) peak at 3350 cm^−1^ assigned to the hydrogen bonded –OH stretching vibrations increased after the addition of PDA, indicating the formation of additional hydrogen bonding by PDA. Taking advantage of evaporation induced self-assembly strategy, conformal dense coating with highly organized layered structure can form from the viscous CNFs/BN/PDA slurry. The slurry with different BN contents is coded to be BCNF10, BCNF30, BCNF50, and BCNF70 for 10 wt %, 30 wt %, 50 wt %, and 70 wt % BN, respectively.

**Figure 2:**
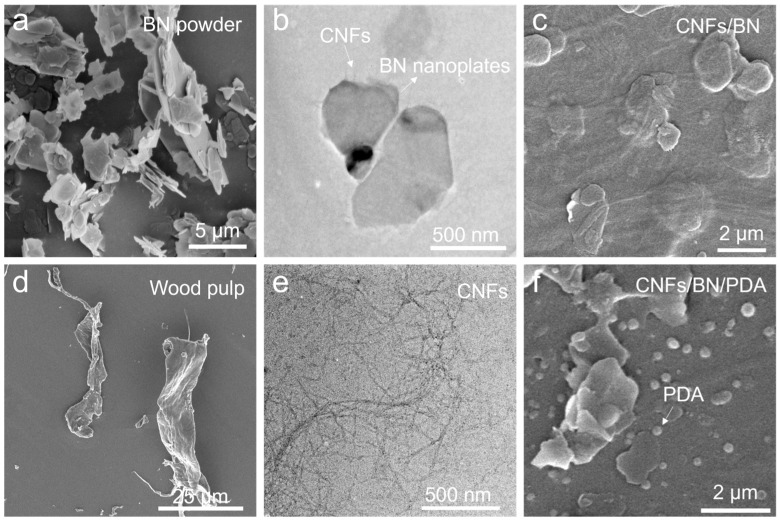
SEM images of pristine BN (**a**) and cellulose fibers (**d**); TEM images of BN (**b**) and CNFs (**e**) after co-exfoliation; SEM images of CNFs/BN mixture before (**c**) and after PDA decoration (**f**).

**Figure 3:**
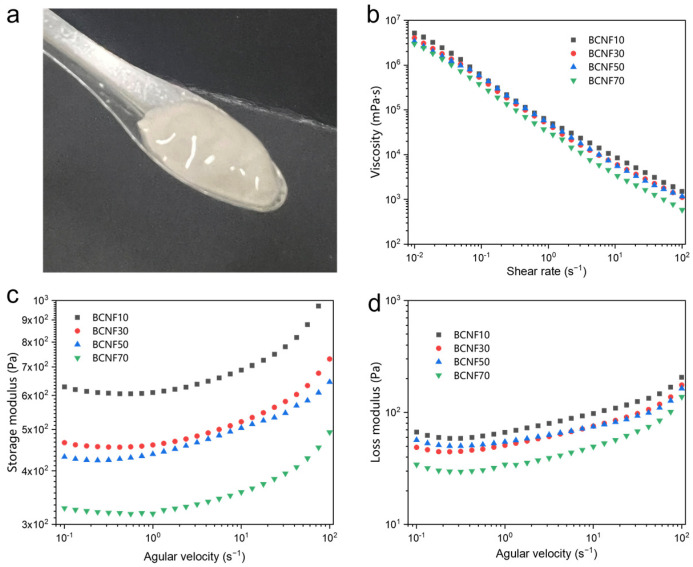
(**a**) The photo of obtained CNFs/BN/PDA slurry (3.5 wt %). (**b**) Viscosities of CNFs/BN/PDA slurries with different BN contents as a function of shear rate. Storage (**c**) and loss (**d**) modulus of CNFs/BN/PDA slurries with different BN contents as functions of frequency.

We investigate the morphological evolution of building blocks during the co-exfoliation and PDA formation. Pristine BN powder consists of nanoplates with a lateral size of 1–10 μm and a thickness of around 200 nm (Figure 2a), while original cellulose fibers from wood pulp have a diameter of around 10–15 μm (Figure 2d). Due to their large size and hydrophobic character, BN powders are difficult to disperse in water. After the pan mill treatment, the BN mixture and cellulose fibers are co-exfoliated into small sized particles to form a stable slurry (Figure 2b,e and Figure 3a), where BN nanoplates are conformal wrapped by an elementary CNFs network with diameter of 3 nm (Figure 2c). We suggest that this co-exfoliation of CNFs/BN dispersion is facilitated by the amphiphilic property of CNFs, which consists of a hydrophobic and hydrophobic crystalline plane [29], while the –OH/-COOH groups of hydrophilic planes assist the dispersion of BN in water [30]. After the addition of PDA, numerous PDA nanoparticles are bonded on the CNFs and BN nanoplates’ surface due to strong adhesion (Figure 2f). This good exfoliation and good dispersion of building blocks is favorable for constructing thermal conductive coating materials.

As shown in Figure 3a, the obtained CNFs/BN/PDA slurry exhibits a homogeneous morphology with good stability up to several months (Figure S3). This is because the amphiphilic character of CNFs allows them to firmly bond on the BN surface, while the high aspect ratio of CNFs forms a physical entanglement network to prevent aggregation through electrostatic repulsion [31]. As is already known, the rheological properties of dispersion play an important role in the coating processability, thus we systematically studied the rheological behavior of the resulting CNFs/BN/PDA slurries with different BN content. In the shear rate–viscosity curves, all the viscosity of slurries shows a similar downward trend with the increase in shear rate, indicating typical shear-thinning non-Newtonian behavior that is favorable for improving processability. This gradual decreased viscosity is caused by the orientation of CNFs/BN along the shear direction under low shear force [32]. Furthermore, with the increase in BN content, the viscoelastic characteristics of the suspensions decrease. The reason for this is that the BN dispersed in the slurry reduces entanglement between CNFs, so the higher the BN content, the less entanglement between the CNFs and the lower viscosity of the slurry [33]. Figure 3c,d show viscoelastic storage modulus (G′) and loss modulus (G″) behavior of CNFs/BN/PDA slurries as a function of angular frequency (ω). For all the slurries, the G′ was much higher than G″ in all of the investigated angular frequency ranges, indicating the formation of a gel-like structure in the slurries. The G′ increases continuously with the decreasing addition of BN content, mainly due to the formation of a more entangled CNFs network with more CNF contents. Additionally, the G′ of slurries has a frequency dependent behavior, where G′ experiences continuously decrease when the frequency is unchanged and increase with the decreasing frequency. This behavior can be explained by the dynamic nanostructure change of slurries. As a high frequency is applied, the initial percolating network of slurries would be broken, leading to the decreased modulus. At relatively low frequency, the destruction and reconstruction of CNFs/BN entanglements can reach a balance at the plateau. Further lowering the frequency provides enough time to significantly reconstruct the percolating network, leading to an increase in elastic and viscous moduli [34].

**Figure 4:**
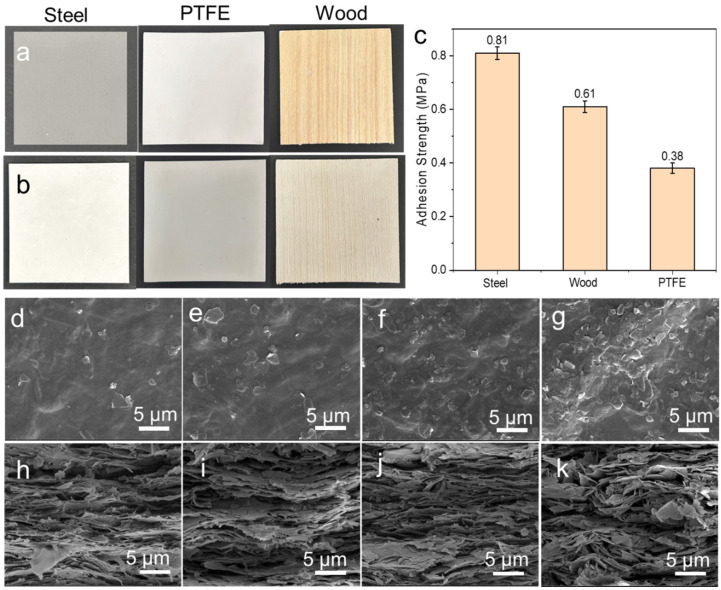
Photos of steel, PTFE and wood before (**a**) and after coating (**b**). (**c**) The adhesion strength of coating on different substrates. SEM images of CNFs/BN/PDA coatings’ morphology. (**d**–**g**) The surfaces of BCNF10, BCNF30, BCNF50, and BCNF70, respectively. (**h**–**k**) Cross-sections of BCNF10, BCNF30, BCNF50, and BCNF70, respectively.

Owing to the good processability and adhesion of the obtained slurry, conformal thermal conductive coating can be applied to various substrate materials. As shown in Figure 4a, the BCNF70 slurries can uniformly coat the smooth steel, polytetrafluoroethylene (PTFE), and rough wood using a simple spray coating technique. The resulting coating exhibits a good smooth surface as illustrated in the 3D topographic images (Figure S4). Also, the aligned texture on the wood is still maintained after the coating treatment, indicating that the coating tightly wraps around the substrate surface. The interface adhesion of the coating was measured to be 0.81, 0.61, and 0.38 MPa for steel, wood and PTFE, respectively, which is good enough to enable the practical applications (Figure 4c). Then, we investigated the structure of the coating with different BN loading. All of the surface has a dense and smooth morphology with uniform BN nanoplates distribution. Increasing BN loading leads to more BN nanoplates being exposed on the coating surface, but they are still wrapped by CNF network to prevent the leakage in the practical applications (Figure S5). For the cross-section, all the coating has a nacre-like layered structure, where BN nanoplates are highly aligned along the substrate surface due to the strong capillary and gravity effect during drying. This uniform layered structure is not only favorable to connect BN nanoplates to form good thermal conductive networks for enhancing thermal conductivity, but also possesses multiple reinforcing mechanisms for mechanical enhancement. Additionally, the CNFs/BN/PDA coating is hydrophilic with a water contact angle of 82.4°. (Figure S6).

**Figure 5:**
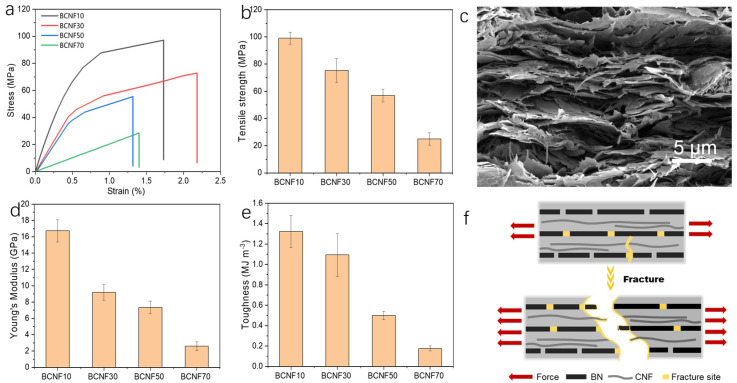
The stress-strain curves (**a**), strength (**b**), Young’s modulus (**d**) and toughness (**e**) of CNFs/BN/PDA composites films with different BN loading (the error bars were obtained from the three sets of data). (**c**) The fractured cross-section SEM image of CNFs/BN/PDA composites. (**f**) The schematic illustration of the fracture mechanism.

Next, the mechanical properties of the composites were investigated since it is critical for service life in practical applications. As illustrated in Figure 5a–d, composites with 10 wt % BN can achieve good mechanical robustness with a strength of 100 ± 4 MPa, modulus of 17 ± 1 GPa and toughness of 1.3 ± 0.2 MJ/cm^3^. The increasing addition of BN nanoplates leads to the decrease in the mechanical properties, but the composites still maintain reasonable strength and toughness, even with 70 wt % BN loading. BN composites usually have limited strength and toughness because of the weak interfacial interactions between BN nanoparticles, while the superstrong CNFs can connect BN together to significantly enhance the interfacial strength [35]. To gain insight into the fracture mechanism of the composites, we investigated the fracture cross-section of the composites (Figure 5c). The SEM image of the cross-section exhibits hierarchical rough layered morphology where many nanofibers and nanoplates are pulled-out. We propose a multiple toughening mechanism response to the mechanical enhancement (Figure 5f). When applying force, the dynamic bonds are broken, followed by the stretching and slipping of CNFs and BN. With further loading, CNFs and BN reorient and align along the loading direction, which will further experience pull-out, delamination, and fracture. This process would largely dissipate fracture energy for achieving good mechanical properties [36].

**Figure 6:**
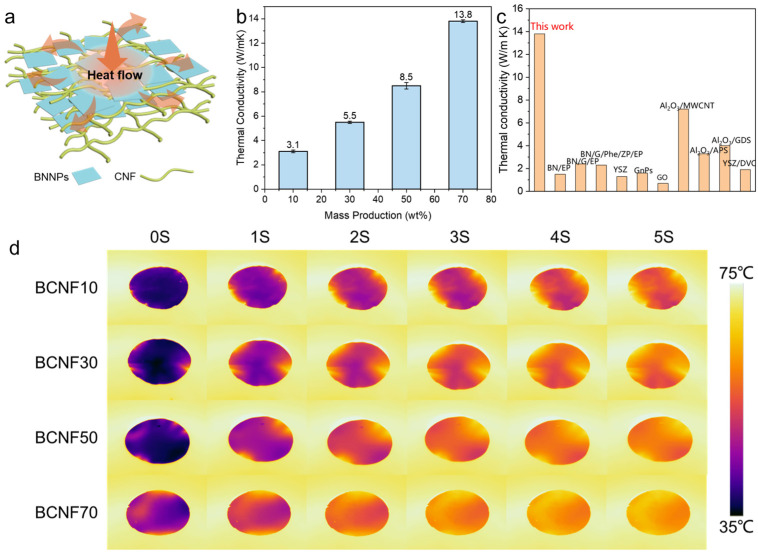
(**a**) Schematic of thermal conduction mechanism. (**b**) Thermal conductivity of CNFs/BN/PDA composites films with different BN loading. (**c**) Comparison of reported thermal conductive composite coatings. (**d**) Infrared thermal images of surface temperature changes of CNFs/BN/PDA composites films placed on an 80 °C hotplate.

The thermal conductivity of the composites coatings is illustrated in Figure 6b. The thermal conductivity of composites with 10 wt % BN loading is around 3.1 W/(m·K), which is superior to most plastics. The thermal conductivity further increases linearly with the increase in BN loading and reaches a high value up to 13.8 W/(m·K) with 70 wt % BN incorporation. We suggest that this high thermal conductivity is owed to the highly ordered thermal conducting network of the composites. These well-exfoliated CNFs and BN nanoplates are closely packed together to form a dense layered structure, where CNFs can work as a thermal conductive bridge to connect BN nanoplates because of its high intrinsic thermal conductivity [37]. Additionally, the smooth and homogenous surface could also reduce the phonon scattering during thermal conduction [23]. The possible mechanism for the high thermal conductivity was schematically illustrated in Figure 6a. The exfoliated BN nanosheets overlapped each other, and the CNFs attached around the BN nanosheets due to hydrophobic–hydrophobic interactions, forming a dense and oriented thermal conductivity network, which minimized the gaps between the BNs, reduced the thermal resistance between the composite interfaces of the BN and CNFs, and improved the thermal properties. When the CNFs/BN were heated, the heat flow diffused rapidly along the network of BN nanosheets and CNFs to the whole, due to the inherent high thermal conductivity, dense thermal network, and low thermal resistance of BN and cellulose, thus exhibiting excellent thermal conductivity [38]. To visually evaluate the heat transfer capability of our coating, we placed the coating films on a hotplate with a temperature of 80 °C. The heat dissipation performance is monitored by the temperature variations through infrared thermal imaging technology (Figure 6d). Obviously, the temperature of composites with higher BN loading increases faster, and can reach 75 °C within just 5 s due to their high thermal conductivity, exhibiting outstanding heat dissipation performance. Additionally, to highlight the heat dissipation performance, we compared the thermal conductivity of our coating materials with other reported thermal conductive coating materials (Table S1) [39,40,41,42,43,44]. As shown in Figure 6b, the thermal conductivity exhibited in this work is far beyond other thermal conductive coating materials. For instance, Xu et al., reported that the addition of 40 wt % BN into epoxy (EP) lead to conductivity of 1.5 W/(m·K), which can further increase to 2.4 W/(m·K) with the addition of graphene. Ligati et al. prepared graphene-loaded paint with a thermal conductivity of 1.6 W/(m·K). Clearly, the thermal conductivity of our work is far higher than these reported materials, even with the same BN loading (Figure S7).

## 4. Conclusions

In summary, we have realized the scalable production of highly thermal conductive CNF/BN/PDA coating by mimicking the layered nanostructure of nacre. Taking advantage of the strong interfacial interaction and the strong mechanical shearing of a pan mill, efficient co-exfoliation and dispersion of CNF and BN can be achieved to produce processible slurry. The addition of PDA can largely increase the adhesion of the slurry to various substrate surfaces by simple spray coating. The resulting coating exhibits nacre-like layered structure with horizontally aligned BN nanoplates that are connected by CNFs. These interconnected networks guarantee ultrafast thermal conductive pathways for phonon transport, as well as multiple reinforcement mechanisms for energy dissipation. As a result, the composites coatings exhibit a high thermal conductivity of 13.8 W/(m·K), which is well beyond most of the previously reported thermal conductive coating, combined with good mechanical properties, low-cost, good adhesion and sustainability. We expect this material will find many real-world applications in the electronic, auto, and aerospace industries.

## Data Availability

Data are available in the main article and Supplementary Materials.

## Supplementary Materials

The following supporting information can be downloaded at: https://www.mdpi.com/article/10.3390/polym16060805/s1, Figure S1. The mechanism of Co-exfoliation/dispersion of BN nanoplate and CNFs. Figure S2. FTIR spectra of BCNF70 and BCNF70 without PDA. Figure S3. (a) The photos of CNFs/BN/PDA slurry. (b) Viscosities of CNFs/BN/PDA slurries. Figure S4. 3D topographic images of BCNF70 on steel, PTFE and wood. Figure S5. SEM image of BCNF70 coating where CNFs (arrows) wrap around BN. Figure S6. Water contact angle of the CNFs/BN/PDA coating. Figure S7. Thermal conductivity comparison as a function of the mass fraction of thermal conductive nanofillers. Table S1. Comparison of thermal conductivity of reported composites coating. Refs. [39,40,41,42,43,44] are cited in the supplementary materials.
