# Supplementary material for: Bioinspired Thermal Conductive Cellulose Nanofibers/Boron Nitride Coating Enabled by Co-Exfoliation and Interfacial Engineering
Source: Polymers (Basel). 2024 Mar 14;16(6):805. doi: 10.3390/polym16060805 (PMC10974445; doi:10.3390/polym16060805)
Supplement: Supplementary file 1 [file polymers-16-00805-s001.zip › polymers-2875345-supplementary.pdf]

Bioinspired Thermal Conductive Cellulose Nanofibers/Boron Nitride Coating Enabled by Co-Exfoliation and Interfacial Engineering

Xinyuan Wan [1], Xiaojian Xia [1], Yunxiang Chen [1], Deyuan Lin [1], Yi Zhou [2] and Rui Xiong [2,*]

1 State Grid Fujian Electric Research Institute, Fuzhou 350007, China; wanxinyuan89103@163.com (X.W.); xia.xiaojian@gmail.com (X.X.); rogerchen614@163.com (Y.C.); lindeyuan_fj@126.com (D.L.)
2 State Key Laboratory of Polymer Materials Engineering, Polymer Research Institute of Sichuan University, Chengdu 610065, China; yizhou@stu.scu.edu.cn
* Correspondence: rui.xiong@scu.edu.cn

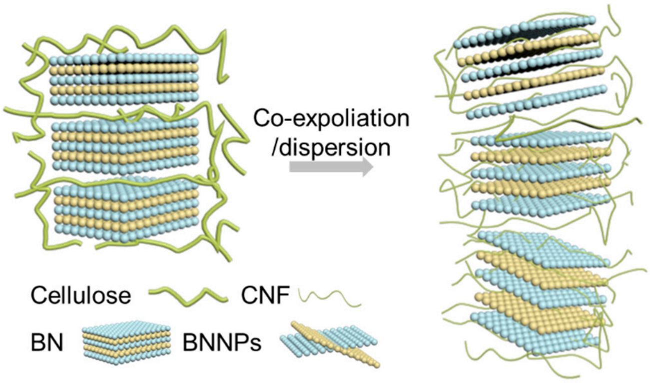

Figure S1. The mechanism of Co-exfoliation/dispersion of BN nanoplate and CNFs.

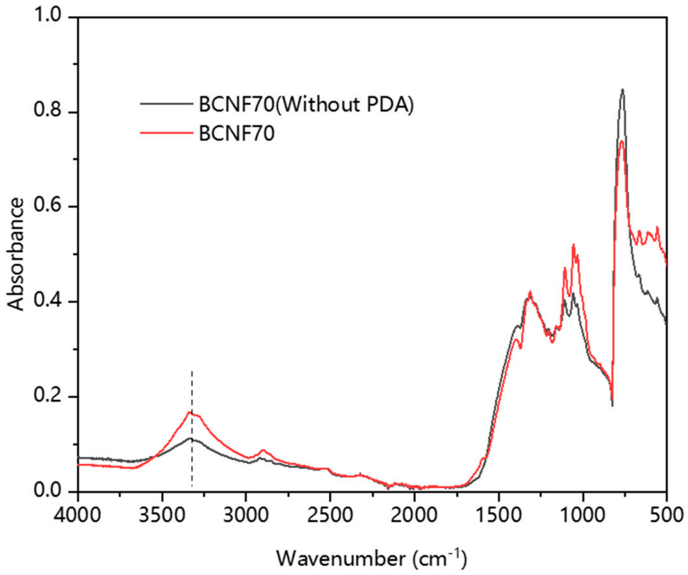

Figure S2. FTIR spectra of BCNF70 and BCNF70 without PDA.

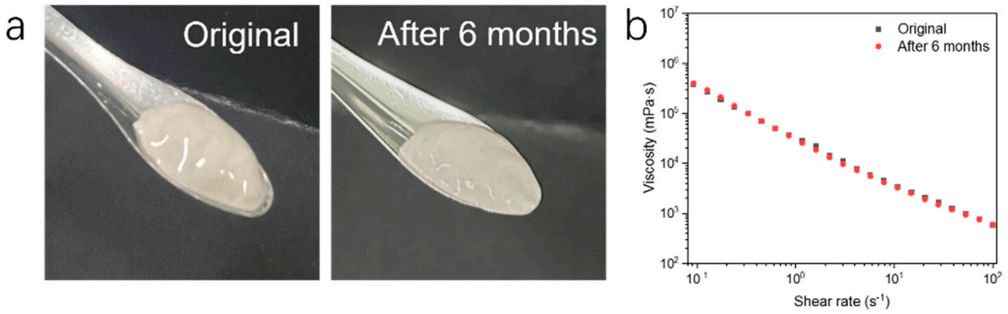

Figure S3. (a) The photos of CNFs/BN/PDA slurry. (b) Viscosities of CNFs/BN/PDA slurries.

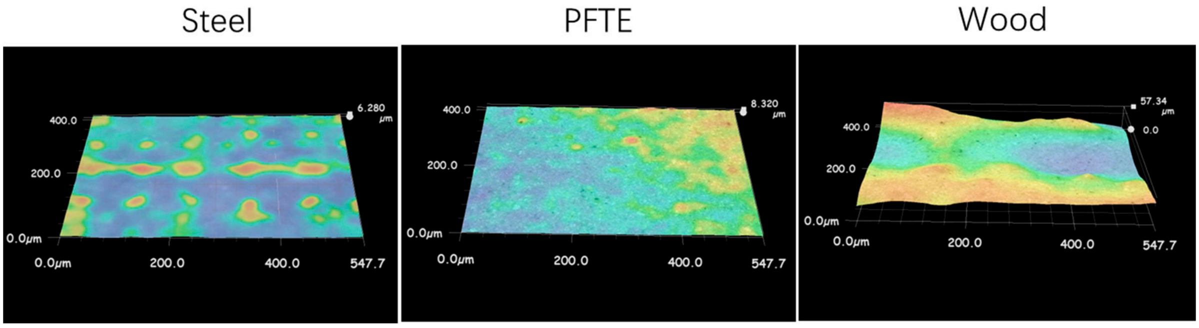

Figure S4. 3D topographic images of BCNF70 on steel, PTFE and wood.

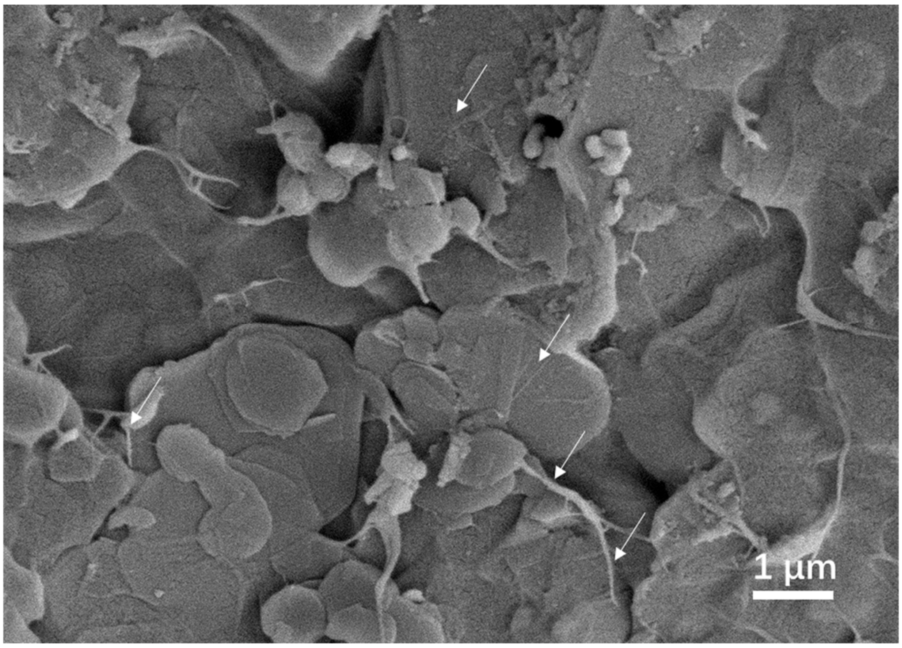

Figure S5. SEM image of BCNF70 coating where CNFs (arrows) wrap around BN.

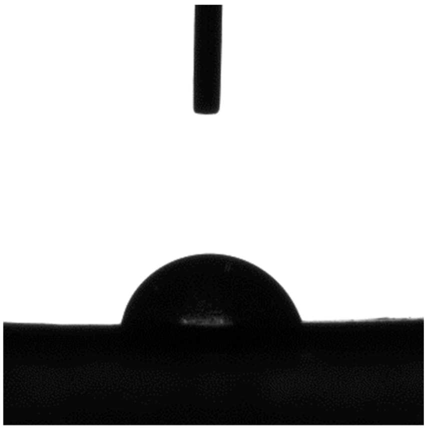

Figure S6. Water contact angle of the CNFs/BN/PDA coating.

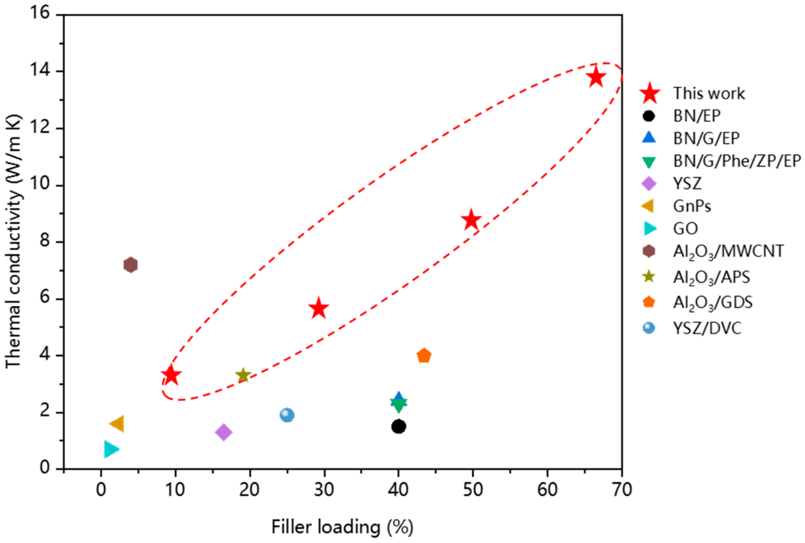

Figure S7. Thermal conductivity comparison as a function of the mass fraction of thermal conductive nanofillers.

Table S1. Comparison of thermal conductivity of reported composites coating.

Materials	Method	Thermal conductivity (W/m K)	Refernces
BN/CNF	TPS	13.8	This work
BN/EP	TPS	1.5	[1]
BN/G/EP	TPS	2.4	[1]
BN/G/Phe/ZP/EP	TPS	2.3	[1]
YSZ	DSC	1.3	[2]
GnPs	TPS	1.6	[3]
GO	TPS	0.7	[3]
Al_2O_3/MWCNT	LFA	7.2	[4]
Al_2O_3/APS	steady-state	3.3	[5]
Al_2O_3/GDS	steady-state	4	[5]
YSZ/DVC	LFA	1.9	[6]

References

1. Xu, F.; Zhang, M.; Cui, Y.; Bao, D.; Peng, J.; Gao, Y.; Lin, D.; Geng, H.; Zhu, Y.; Wang, H. A Novel Polymer Composite Coating with High Thermal Conductivity and Unique Anti-Corrosion Performance. *Chemical Engineering Journal* **2022**, *439*, 135660, doi:10.1016/j.cej.2022.135660.
2. Chi, W.; Sampath, S.; Wang, H. Ambient and High-Temperature Thermal Conductivity of Thermal Sprayed Coatings. *Journal of Thermal Spray Technology* **2006**, *15*, 773–778, doi:10.1361/105996306X146730.
3. Ligati, S.; Ohayon-Lavi, A.; Keyes, J.; Ziskind, G.; Regev, O. Enhancing Thermal Conductivity in Graphene-Loaded Paint: Effects of Phase Change, Rheology and Filler Size. *International Journal of Thermal Sciences* **2020**, *153*, 106381, doi:10.1016/j.ijthermalsci.2020.106381.
4. Bakshi, S.R.; Balani, K.; Agarwal, A. Thermal Conductivity of Plasma - Sprayed Aluminum Oxide—Multiwalled Carbon Nanotube Composites. *Journal of the American Ceramic Society* **2008**, *91*, 942–947, doi:10.1111/j.1551-2916.2007.02081.x.
5. Shakhova, I.; Mironov, E.; Azarmi, F.; Safonov, A. Thermo-Electrical Properties of the Alumina Coatings Deposited by Different Thermal Spraying Technologies. *Ceramics International* **2017**, *43*, 15392–15401, doi:10.1016/j.ceramint.2017.08.080.
6. Curry, N.; VanEvery, K.; Snyder, T.; Markocsan, N. Thermal Conductivity Analysis and Lifetime Testing of Suspension Plasma-Sprayed Thermal Barrier Coatings. *Coatings* **2014**, *4*, 630–650, doi:10.3390/coatings4030630.
